# Speed Matters: Challenging the Notion of Velocity-Independent Rigidity Using Technological Devices in People with Parkinson’s Disease: A Systematic Review

**DOI:** 10.3390/neurolint17110186

**Published:** 2025-11-17

**Authors:** Roberto Cano-de-la-Cuerda, Cecilia Estrada-Barranco, Patricia Martín-Casas, Selena Marcos-Antón, Rosa María Ortiz-Gutiérrez, Sofía Laguarta-Val, Carmen Jiménez-Antona

**Affiliations:** 1 Department of Physical Therapy, Occupational Therapy, Physical Medicine and Rehabilitation, Faculty of Health Sciences, Rey Juan Carlos University, 28922 Alcorcón, Spain; roberto.cano@urjc.es (R.C.-d.-l.-C.); carmen.jimenez@urjc.es (C.J.-A.); 2 Physiotherapy Department, Faculty of Health Sciences, Universidad Europea de Madrid, Villaviciosa de Odón, 28670 Madrid, Spain; 3 InPhysio Research Group, Physiotherapy Department, Nursing, Physiotherapy and Podiatry Faculty, Health Research Institute of the Hospital Clínico San Carlos (IdISSC), Complutense of Madrid University, 28040 Madrid, Spain; pmcasas@ucm.es (P.M.-C.); rosaorti@ucm.es (R.M.O.-G.); 4 Department of Health Sciences, Universidad Villanueva, 28034 Madrid, Spain

**Keywords:** Parkinson’s disease, rigidity, velocity-dependence, velocity-independence, biomechanical assessment, muscle tone, objective assessment, passive movement, quantitative evaluation, technological devices

## Abstract

**Objectives**: The application of well-controlled, quantitative measurement systems has challenged the traditional notion that rigidity in Parkinson’s disease (PD) is a velocity-independent phenomenon. This review aimed to evaluate whether rigidity in PD is velocity-dependent or velocity-independent across different joints, body regions, testing speeds, and methodologies. **Methods**: This systematic review followed the Preferred Reporting Items for Systematic reviews and Meta-Analyses (PRISMA) guidelines. Methodological quality of cross-sectional studies was assessed using the Appraisal Tool for Cross-Sectional Studies (AXIS), and reporting completeness was evaluated with the Strengthening the Reporting of Observational Studies in Epidemiology (STROBE) checklist. **Results**: Seventeen studies were included and analyzed by the body part assessed (wrist, elbow, hand, knee, trunk). Rigidity quantification in PD used various biomechanical technologies, sometimes combined with neurophysiological methods. Although rigidity is classically considered velocity-independent, experimental evidence suggests a more complex behavior, partially velocity-dependent, especially at moderate to high angular velocities. Methodological quality was variable but generally acceptable, with more recent studies showing stronger adherence to AXIS. However, compliance with STROBE reporting standards remained inconsistent. **Conclusions**: While rigidity in PD has not been classically defined as velocity-dependent, current evidence indicates that, under specific testing conditions, rigidity increases with passive movement velocity. These findings challenge traditional clinical assumptions and emphasize the need for standardized measurement protocols.

## 1. Introduction

Classically, pathological hypertonia has been categorized as either spasticity or rigidity, based on the muscle’s response to varying speeds of assessment. Spasticity, on one hand, is a sensorimotor disorder that affects approximately 85% of individuals with multiple sclerosis [1], 65–78% of people with stroke [2], and 65–78% of individuals with spinal cord injury [3]. The most widely accepted and frequently cited definition of spasticity originates from Lance, who described it as follows: “spasticity is a motor disorder characterized by a velocity-dependent increase in tonic stretch reflexes (muscle tone), with exaggerated osteotendinous reflexes, which results from the hyperexcitability of the stretch reflex and is one of the components of the upper motor neuron syndrome” [4]. Subsequently, the Support Programme for Assembly of a Database for Spasticity Measurement (SPASM) provided an expanded definition, describing spasticity as “an altered sensory-motor control, due to an upper motor neuron lesion and presented as an intermittent or maintained involuntary muscle activation” [5]. The most notable aspect of this new definition is its emphasis on the role of sensory-motor alterations as the underlying cause of involuntary and inappropriate muscular activity. It also notably omits the velocity-dependent phenomenon and the mention of the tonic stretch reflex. More recently, Van den Noort et al. [6] attempted to establish a clear and consistent terminology to describe and measure the pathophysiological neuromuscular response to passive muscle stretch. This consensus paper proposed to use the term hyper-resistance to describe the phenomenon of altered neuromuscular response during passive stretching, rather than, for example, “spasticity” or “hypertonia”. They indicated that it is essential to distinguish non-neural (tissue-related) from neural (central nervous system-related) contributions to hyper-resistance.

In contrast, rigidity has been classically defined as an increase in muscle tone which is velocity-independent. Characterized by either a lead-pipe or cogwheel pattern (sometimes accompanied by an underlying tremor), rigidity is one of the hallmark symptoms of Parkinson’s disease (PD) [7], affecting up to 89% of individuals [8]. A significant reduction in dopamine levels in the basal ganglia has been strongly associated with the development of both akinesia and rigidity [9]. However, there is ongoing debate regarding the precise pathophysiological mechanisms underlying rigidity, particularly concerning the relative contributions of different factors to its clinical presentation, including the contribution of neural and non-neural components to hypertonia. Some researchers attribute the primary abnormalities to the neural component, including excessive monosynaptic stretch reflexes, long-latency stretch reflexes, the emergence of a tonic stretch reflex, and the development of a shortening reaction as possible mechanisms [10]. Others highlight the influence of an altered frequency in the discharge of neurons from the subthalamic nucleus and the alteration of the connectivity between the networks that link the cerebellum, the motor cortices, and temporal, occipital, and caudate nuclei in individuals with moderate PD [9]. Other authors, as mentioned above, have recognized that hypertonia not only concerns neural aspects but also the biomechanical properties of the muscle being tested and surrounding soft tissue (collectively known as the non-neural component of muscle tone, part of which is velocity-dependent) [6].

The clinical assessment of rigidity in individuals with PD relies on a semi-quantitative scoring system, commonly using clinical scales such as the Unified Parkinson’s Disease Rating Scale (UPDRS) [5]. Rigidity is evaluated through slow passive movements of major joints while the individual remains in a relaxed position, with the examiner manipulating the limbs and neck. Initially, the assessment is performed without employing any activation maneuvers. The neck and each limb are individually tested and rated. In cases where no rigidity is detected, it is recommended to employ an activation maneuver (e.g., Froment’s maneuver), which involves tasks such as finger tapping, fist opening and closing, or heel tapping in a limb not being tested. Scores are given in a 5-point ordinal scale, from 0 = Normal: No rigidity; up to 4 = Severe: Rigidity detected without the activation maneuver and full range of motion not achieved [5]. However, even a neurologist with expertise in movement disorders can commit an error of up to 20% in the accuracy of the diagnosis [11,12] and notably, the use of different speeds during examination is not routinely considered, even though several authors have expressed doubts about the non-velocity-dependent nature of rigidity in PD [13,14,15,16].

In this context, biomechanical objective methods have been developed to quantify muscle tone abnormalities with fine-grained precision and control. They could be potentially useful for assessing rigidity in PD, improving our understanding of the nature of muscle response to different speed conditions [12]. Biomechanical assessment of spasticity provides highly reproducible data, offering valuable insights for both research purposes and treatment evaluation. The main advantage of this methodology lies in its ability to deliver a more accurate analysis of passive movement compared to traditional clinical scales [17].

One of these methods involves using servomotors, which mobilize a body segment at a desired speed. When this speed is constant throughout the range of motion (ROM) mobilized, this is known as isokinetic dynamometry. This technique allows for the collection of information relative to the offered resistance as an objective measure of muscle tone [12,15], both for the neural component of hypertonicity, through proper selection of evaluation speeds, plus the non-neural (viscoelastic) component of the muscle response to stretch, as both contribute to the presence of rigidity. Neurophysiological assessment, which often complements biomechanical evaluation, involves recording muscle electrical activity using electromyography, which allows for the detection of alterations in muscular tone and the definition of the underlying physiological mechanisms. Several techniques are available for this purpose, all based on measuring the reflex response of the neuromuscular system to an elicited stimulus. The most used stimuli for spinal reflex elicitation include electrical, mechanical, proprioceptive, and cutaneous modalities [17]. Particularly characteristic in PD is the study of the cycle of recuperation of the H reflex, the recurrent inhibition, the F wave, or the polysynaptic skin reflexes, the values of which allow the identification of the presence of an alteration of the tone and the related modality [11].

In this line, the use of these well-controlled, quantitative, and accurate systems has challenged the traditional notion that stiffness is a velocity-independent phenomenon, as reported in scientific literature in people with PD [12,18]. Therefore, the objective of this systematic review is to evaluate whether rigidity in people with PD is a velocity-dependent or velocity-independent phenomenon, with a particular focus on the muscle tone behavior across different joints and body regions, considering different testing speeds and measurement methodologies.

## 2. Materials and Methods

### 2.1. Search Strategy

This systematic review was conducted in accordance with the Preferred Reporting Items for Systematic Reviews and Meta-Analyses (PRISMA) [19] (Supplementary Materials Table S1). The review protocol was prospectively registered in the International Prospective Register of Systematic Reviews (PROSPERO) under registration number CRD420251107741.

A comprehensive literature search was conducted by two reviewers across these electronic databases: PubMed, Scopus, Web of Science, IEEE Xplore, Cochrane, PEDro and ACM Digital Library, covering all publications from database inception until 31st July 2025. Key words used were “rigidity”, “hypertonia”, and “muscle stiffness”, crossed with “evaluation”, “assessment”, “quantification”, “Parkinson”, and “parkinsonian”, utilizing pertinent Boolean terms and specific search filters from each database ([“rigidity” OR “hypertonia” OR “muscle stiffness”] AND [“evaluation” OR “assessment” OR “quantification”] AND [“Parkinson” OR “parkinsonian”]). The search strategy was developed using a combination of controlled vocabulary (MeSH terms) and free-text keywords, applied according to the indexing options available in each database.

Two authors independently searched data and analyzed abstracts and titles to decide if a study met the eligibility criteria. In case of disagreement, this was solved by a third independent author.

### 2.2. Study Selection

Studies were eligible if they met the following criteria: (i) included people with PD with no age limit; (ii) used objective measures for rigidity assessment; (iii) reported muscle tone responses at different speed assessments; (iv) were published in English. This systematic review excluded articles according to the following exclusion criteria: (i) studies published as study protocols, theoretical papers and clinical trials registration; (ii) studies that did not use technology or objective assessment methods (iii) systematic or no systematic reviews.

### 2.3. Data Extraction and Analysis

A systematic review was conducted by two independent authors (R.C.-d.-l.-C. and C.J.A.) which screened titles and abstracts against the eligibility criteria. Full texts of potentially relevant articles were obtained and assessed independently by the same reviewers. All retrieved records were imported into a reference management software, and duplicates were removed.

We used a standardized data extraction protocol, collecting information about population, assessment methods and results. Also, for each study we defined the sample size, joint explored, type of system used for the quantification of rigidity, evaluation protocol and rigidity outcome measures.

Finally, both authors reached an agreement about each extracted data item. Any disagreements that arose were resolved by a third, independent author (S.L.V.). Articles retrieved through reference lists were also considered.

### 2.4. Assessment of the Methodological Rigor of the Included Studies

The methodological quality of the included cross-sectional studies was evaluated using the Appraisal Tool for Cross-Sectional Studies (AXIS) [20]. This validated tool is specifically designed for the critical appraisal of cross-sectional studies in health research. It consists of 20 items assessing key domains such as study design, sampling strategy, measurement validity, statistical analysis, and risk of bias. Two independent reviewers (R.C.-d.-l.-C. and C.J.A.) applied the AXIS tool to each included study. Discrepancies in scoring were resolved through discussion or consultation with a third reviewer (S.L.V.) when necessary. Each item was rated as “Yes,” “No,” or “Don’t know,” following the standardized AXIS checklist. A narrative synthesis of methodological strengths and limitations was provided for each study. While the AXIS tool does not provide a cumulative score, we considered studies with multiple “No” responses in critical domains—such as sampling bias, measurement reliability, or inadequate statistical reporting—as having lower methodological quality. These assessments informed the interpretation of results and the overall strength of the evidence.

In parallel, the Strengthening the Reporting of Observational Studies in Epidemiology (STROBE) checklist was used to evaluate the completeness and transparency of reporting. This checklist comprises 35 items covering essential components of observational research reports, including title, abstract, study design, participants, data sources, statistical methods, and results presentation [21]. Each item is scored by “yes”, “no”, or “unclear”, so that “yes” is equivalent to 1 point and “no” or “unclear” are equivalent to 0 points. Thus, high scores indicate greater degrees of confidence or reliability of information provided by the authors.

## 3. Results

**Figure 1:**
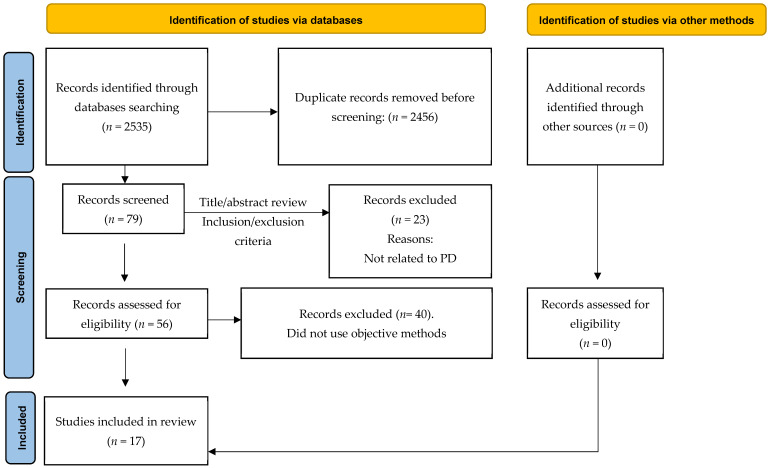
Flow diagram of the search strategy results.

A description of the selection process is described in Figure 1. Initial searches generated a total of 2535 results, of which 79 were potential studies. After applying eligibility criteria, 17 studies [13,14,15,16,22,23,24,25,26,27,28,29,30,31,32,33,34] were finally included (Figure 1).

### 3.1. Sample Characteristics

The reviewed studies included a total of 368 individuals with PD, with sample sizes ranging from 4 to 47 patients and, in many cases, healthy control groups [13,15,16,22,24,25,27,28,29,30,33] for comparison. A total of 146 healthy subjects were recruited. Additionally, two studies [12,32] enrolled 28 stroke patients, with sample sizes of 12 and 14, respectively. Clinical characteristics varied across studies, with most reporting age, disease duration, and Hoehn and Yahr (H&Y) staging. Not all studies reported UPDRS scores and medication states (ON/OFF).

While a few studies lacked detailed clinical data, others provided comprehensive profiles, including rigidity sub-scores and functional assessments, reflecting a heterogeneous but clinically relevant PD population (Table 1).

### 3.2. Participant Characteristics by Limb or Anatomical Region Explored

Eight studies focused on the assessment of wrist tone [13,22,23,24,25,26,27,28] (Table 1): Sample sizes ranged from 4 to 25 participants per PD group, with control groups included in most studies (*n* = 10–25). Patients’ ages ranged from approximately 50 to 73 years, with several studies reporting mean ages. Disease duration among participants varied from 1 to 30 years. Hoehn & Yahr (H&Y) stages were inconsistently reported, spanning from stage 0 to 5 when available. UPDRS rigidity scores ranged from 0 to 3 in the ON and OFF medication states, with some studies specifying upper limb rigidity sub-scores (e.g., 2.1 ± 0.8). Several studies also reported testing in both medicated (ON) and unmedicated (OFF) conditions. Control groups were age- and sex-matched where specified. No additional demographic or clinical stratification was provided in some cases (e.g., Zito et al. [28]). One study did not include a control group (Powell et al. [24]).

Four studies focused on the assessment of elbow and hand [14,16,29,32] (Table 1): Sample sizes for PD groups ranged from 16 to 47 individuals. Control groups were included in most studies (*n* = 12 to 22), with some also incorporating stroke or hemiparesis patients for comparative analysis. Participant ages varied widely, with reported means ranging from approximately 60.6 to 74.4 years for PD patients. In Lee et al. [14], 16 individuals with rigid parkinsonism had an age range of 27–85 years (mean 60.6 ± 13.5), and in Endo et al. [29] the PD group had a mean age of 74.4 ± 6.2 years. Huang et al. [32] reported the PD group’s mean age as 68.3 ± 9.9 years. Clinical classification or scoring was inconsistently reported. For example, Endo et al. [29] detailed UPDRS rigidity scores: 8 patients scored 1, 9 scored 2, and 3 scored 3. Rothwell et al. [16] categorized PD patients into four groups based on the degree of elbow rigidity or tremor, without specifying UPDRS metrics. All PD participants in Endo et al. [29] were assessed while on medication. Some studies (e.g., Lee et al. [14], Huang et al. [32]) included additional neurological cohorts (e.g., stroke, spastic hemiparesis) to contrast tone profiles with those of parkinsonian patients.

Three studies focused on the assessment of knee and ankle [30,31,33] (Table 1): Two studies investigated knee joint rigidity in PD using objective methods. Sample sizes included 10 PD patients and 10 controls in Nuyens et al. [30], and 40 PD patients in Uslu et al. [31]. In Nuyens et al. [30], people with PD had a mean age of 65.4 ± 7.41 years and an average disease duration of 10 ± 5.23 years. Modified Hoehn and Yahr (H&Y) stages ranged from 2 to 5, and Schwab and England scores varied between 20% and 90%. In Uslu et al. [31], the average disease duration was 5.5 ± 0.67 years. H&Y stages ranged from 1 to 4. Patients were stratified into four subgroups according to the UPDRS: UPDRS 1 (*n* = 8; 4 male, 4 female), UPDRS 2 (*n* = 16; 11 male, 5 female), UPDRS 3 (*n* = 11; 5 male, 6 female), and UPDRS 4 (*n* = 5; 2 male, 3 female). No control group was reported for this study. The study by Gregoric et al. [33] focused on ankle assessment included a sample of seven patients diagnosed with PD. No more clinical information was provided.

Regarding the selected studies focusing on trunk assessment [15,34] (Table 1), two studies focused on this topic. The study by Mak et al. [15] included 15 people with PD and 15 healthy controls. The PD group had a mean age of 64.7 years (SD = 8.7), with H&Y staging scores ranging from 2 to 3. The study by Cano-de-la-Cuerda et al. [34] involved 36 people with PD. The mean age was 62 years (SD = 11). According to the H&Y staging, 24 patients were classified as stage II, 8 as stage IB (unilateral and axial involvement), and 4 as stage III. The mean UPDRS III score was 22 (SD = 8), and the mean disease duration was 55.4 months (SD = 14.3). Functional status, measured by the Schwab and England scale, showed that 26 patients reached 80%, 7 reached 90%, and 3 reached 100%.

### 3.3. Objective Methods and Protocols Employed

A wide range of objective technologies were employed to quantify rigidity in Parkinson’s disease, predominantly electromechanical devices, isokinetic dynamometry, and biomechanical systems often combined with surface electromyography (EMG). These methods were applied across various joints—wrist, elbow, knee, trunk, and ankle—using passive movements at controlled angular velocities. Most studies demonstrated a velocity-dependent increase in rigidity, with higher movement speeds eliciting greater resistive torque and reflex activity. Devices such as the NeuroFlexor [25] and pendulum-based systems [31,32] provided refined assessments of neural versus non-neural contributions to rigidity. Overall, these technologies enabled precise, reproducible measurements and revealed correlations with clinical severity, medication state, and disease progression.

The reviewed studies employed a wide range of passive movement protocols across different joints—primarily the wrist, elbow, knee, trunk, and ankle—to assess rigidity in PD. Angular velocities varied substantially, from very slow (5°/s) [25] to extremely fast (up to 600°/s) [16], with several studies applying multiple velocities to explore the velocity-dependence of rigidity. Movements were typically sinusoidal or ramped, with angular displacements ranging from ±15° to ±45°, and in some cases up to 90°. Protocols often included randomized or sequential velocity conditions, and some incorporated both “on” and “off” medication states. The diversity in protocols reflects efforts to optimize sensitivity and specificity in detecting rigidity-related biomechanical and neurophysiological responses.

### 3.4. Main Findings Regarding Velocity-Dependent Versus Velocity-Independent Responses

Most studies reviewed reported a velocity-dependent component of rigidity in PD. Increased angular velocities during passive joint movements—particularly at the wrist, elbow, knee, and trunk—were consistently associated with greater resistive torque, angular impulse, or reflex activity (Table 1).

Several highlighted that higher velocities enhanced the sensitivity of rigidity detection and correlated with clinical severity (Table 1). Most studies questioned the traditional notion of rigidity being velocity-independent, suggesting that rigidity may share velocity-dependent features with spasticity.

### 3.5. Assessment of Methodological Quality of the Studies

The methodological quality of the included cross-sectional studies was appraised using the AXIS tool, comprising 20 items assessing study design, reporting, and bias risk [20]. Overall, the quality of the studies varied, with more recent studies generally demonstrating higher adherence to AXIS criteria (Table 2).

All studies clearly stated their objectives and utilized appropriate methods for data collection. However, several earlier studies lacked explicit sample size justification and did not report non-responders, limiting assessment of selection bias. Most studies adequately described the target population and employed valid outcome measures. Nonetheless, a few did not clearly report on measurement reliability or validity.

Participant selection was generally well-defined, though some studies failed to provide sufficient detail on recruitment or inclusion/exclusion criteria. Reporting of statistical methods was mostly adequate, yet some studies omitted details on handling missing data or potential confounders.

Most studies provided clear results with appropriate descriptive statistics. However, some lacked sufficient detail regarding non-responders or response rates, potentially introducing bias. More recent investigations consistently met nearly all AXIS criteria, reflecting improvements in study design and reporting standards. In contrast, earlier studies (e.g., Teräväinen et al. 1989 [13]; Rothwell et al. 1983 [16]) showed deficiencies in key areas such as sample size justification, non-responder analysis, and clarity of inclusion criteria. In summary, while methodological rigor varied among included studies, the overall quality was sufficient to support the synthesis of findings, with confidence in the more recent and comprehensively reported works.

Assessment of the 17 included studies using the STROBE checklist (comprising 22 items and 35 sub-items) revealed variable adherence to reporting standards (Table 3). Most studies adequately described their background and objectives, with consistent reporting of study design (Item 4), rationale (Item 2), and clearly defined outcomes and variables (Item 7). However, only a minority fully reported on sample size calculations (Item 10), efforts to mitigate bias (Item 9), or detailed statistical methods related to confounding, subgroup analyses, and sensitivity analyses (Items 12a–e). Data on participant flow (Items 13a–c) and missing data (Items 14b, 16b) were often incomplete or absent. Although outcome data and key results were generally well reported (Items 15, 18), limitations (Item 19) and discussions on generalizability (Item 21) were frequently underreported or superficially addressed. Funding disclosures (Item 22) were missing in over half of the studies. Only one recent study (Asci et al., 2023) demonstrated comprehensive adherence across nearly all STROBE items [21]. These findings underscore the need for improved methodological transparency and standardization in the reporting of observational studies evaluating rigidity in PD.

### 3.6. Strengths, Weaknesses, Opportunities and Threats (SWOT) Analysis of the Included Studies

A comprehensive SWOT analysis was conducted to summarize the main findings of the current revision and propose new areas for exploration (Table 4).

## 4. Discussion

The primary objective of this systematic review was to determine whether rigidity in people with PD exhibits velocity dependency, that is, whether an increase in the speed of passive joint movement leads to an increase in muscle tone, or not. Clarifying this issue has substantial clinical and neurophysiological implications, particularly in differentiating rigidity from spasticity and refining quantitative assessments and treatments in a key motor symptom in PD.

Parkinsonian rigidity is defined by a consistent and uniform resistance to passive limb movements, which appears largely unaffected by the speed or direction of movement. Clinical features such as the cogwheel effect and reinforcement during specific maneuvers further characterize this phenomenon [30]. Nonetheless, rigidity is notably variable, influenced by a range of physiological inputs including proprioceptive feedback, central nervous system modulation, and the patient’s medication status, as well as motor fluctuations typical of PD. These intrinsic fluctuations pose challenges for accurate and reproducible measurement, a difficulty compounded by methodological inconsistencies such as differences in testing devices and examiner technique. In particular, reliance on manual assessment tools can introduce variability, highlighting the potential benefits of motorized devices that deliver standardized movement patterns for more objective evaluation. Moreover, repetitive passive movements appear to affect rigidity, a clinically observed yet under-investigated effect that may hold therapeutic implications [35]. The precise pathophysiological mechanisms behind rigidity remain unresolved; while reflex pathways have been implicated, contrasting findings suggest that changes in the muscle’s inherent mechanical properties also contribute. This complexity underscores the importance of incorporating electromyographic analysis in protocols examining muscle responses to stretching in PD [36].

The seminal study by Rothwell et al. [16] is one of the earliest empirical references suggesting that rigidity in PD is not velocity-dependent. This conclusion was drawn from neurophysiological experiments assessing the behavior of the long-latency stretch reflex (LLSR) in PD patients. The authors reported that, unlike in spasticity, the amplitude of the LLSR did not significantly increase with faster passive joint movements, leading to the inference that parkinsonian rigidity may be fundamentally distinct from spastic hypertonia in its lack of velocity dependence. While this study was pioneering in its neurophysiological approach, particularly in distinguishing rigidity from spasticity, several methodological limitations substantially constrain the generalizability of its conclusions. Most notably, the velocity of passive stretch was not objectively quantified. Movements were manually applied and broadly categorized as “slow” or “fast,” without precise measurement in degrees per second, thereby precluding standardized comparisons across participants or across studies. Additionally, mechanical resistance (e.g., torque, work) was not measured, which limits the ability to assess rigidity from a biomechanical standpoint. Moreover, the experimental setup focused exclusively on electrophysiological outputs (EMG recordings), without integrating complementary biomechanical metrics or modeling of muscle stiffness. This is a critical limitation, as more recent evidence has demonstrated that rigidity arises from both neural (reflex-mediated) and non-neural (viscoelastic) components, which cannot be fully captured through EMG analysis alone. This is a critical limitation, as more recent evidence has demonstrated that rigidity arises from both neural (reflex-mediated) and non-neural (viscoelastic) components that may be associated with rigidity as the PD progresses, which cannot be fully captured through EMG analysis alone.

Another significant shortcoming lies in the limited clinical characterization of the PD sample. The study did not report participants’ H&Y stages, disease duration, or medication status, nor were standardized motor assessments (e.g., UPDRS) included. This lack of clinical context restricts interpretability, as rigidity severity and velocity responsiveness may vary substantially depending on disease stage and dopaminergic state.

In contrast, more recent studies have leveraged advanced technologies to measure rigidity under well-controlled and reproducible conditions [13,14,16,22,23,24,25,26,27,28,29,30,31,32,33,34]. These approaches have employed defined angular velocities and simultaneous EMG-kinematic recordings to quantify the velocity-rigidity relationship. Multiple studies have demonstrated that rigidity often shows a partial or clear increase with stretch velocity, particularly at distal joints, challenging Rothwell’s original assertion. In summary, although Rothwell et al. [16] provided an important conceptual distinction between rigidity and spasticity, the methodological limitations—namely, imprecise control of movement velocity, absence of torque quantification, and insufficient clinical characterization—undermine the strength of their conclusion regarding velocity independence. The current body of literature, built on more rigorous experimental frameworks, increasingly supports the notion that rigidity in PD can exhibit velocity-dependent behavior, depending on the biomechanical and neurophysiological context in which it is evaluated.

Our research group had previously reviewed the quantitative methods used to evaluate rigidity in PD [12]. The papers included utilized servomotor systems to passively mobilize joints at controlled speeds and amplitudes while quantitatively measuring biomechanical responses such as torque, force, and angular displacement. Seventeen studies were included, predominantly investigating the wrist and elbow, with others examining the thumb, knee, cervical spine, and trunk. Despite anatomical differences, protocols generally standardized subject positioning, isolated joint movement, defined ranges of motion (ROM), applied variable speeds (typically slow and fast), controlled rest periods, and repeated measures. For wrist rigidity assessment, optimal detection occurs within a 60–90° ROM and speeds between 140–190°/s [13,24,27,28]. Contrary to classical definitions, a previous review [12] showed that rigidity was found to be speed-dependent, with low sensitivity below 70°/s and reflex saturation above 300°/s [13,16]. A wider ROM and faster speeds enhanced test sensitivity and correlated better with clinical scales. Elbow studies showed exploration ROM between 40° and 70°, with torque and reflex responses significantly elevated in Parkinson’s patients compared to controls [12]. However, there remains no consensus on the relationship between disease severity and stretch reflex intensity, nor on how dopaminergic medication influences rigidity, due to inconsistent findings. Additional studies highlighted the importance of stretch speed on reflex magnitude, as seen in the thumb’s interphalangeal joint, and demonstrated combined electromyography and mechanical assessments to reveal increased coactivation of muscles around the knee during stretching in people with PD [12]. Investigations of the cervical spine and trunk showed increased peak torque and resistance at the end of ROM in PD subjects, with difficulties in muscle relaxation post-stretch, the greatest resistance obtained at the end of the ROM in flexion and extension movements at different speeds and strong correlations between rigidity and clinical variables [37].

In our review, a diverse range of technologies and measurement protocols was employed across the included studies. Electromechanical devices such as isokinetic dynamometers, torque-controlled manipulanda, pendulum-based systems, and the NeuroFlexor were commonly used to capture biomechanical and neurophysiological parameters. These tools varied in complexity and sensitivity, with more recent studies integrating EMG and force sensors to distinguish between neural (e.g., reflex-mediated) and non-neural (e.g., viscoelastic) components of rigidity.

Protocols for passive movement assessment differed significantly, particularly with respect to joint targeted, angular velocities tested, patient positioning, and medication state. Velocities ranged from very slow (5°/s) to extremely fast (up to 600°/s). Movements were assessed with angular displacements ranging from ±15° to ±45°, and in some cases up to 90°. Protocols often included randomized or sequential velocity conditions, and some incorporated both “on” and “off” medication states. The collective findings from the reviewed studies provide compelling evidence that parkinsonian rigidity exhibits a velocity-dependent component. Across multiple experimental paradigms—ranging from electromechanical devices to isokinetic dynamometry and pendulum tests—rigidity consistently increased with higher angular velocities of passive joint movement. This trend was observed in various joints (wrist, elbow, knee, trunk, ankle) and across different measurement techniques (e.g., torque, angular impulse, EMG activity). This increase in resistance could be interpreted as a facilitation maneuver. This possibility will be considered as a hypothesis to be further explored in future studies. Notably, studies such as those by Teräväinen et al. [13], Asci et al. [23], Powell et al. [24], and Mak et al. [15] demonstrated a clear amplification of resistance or reflex activity at higher movement speeds. Furthermore, neurophysiological data (e.g., long-latency reflexes) also scaled with velocity, reinforcing the biomechanical observations. While some authors as Lee et al. [14] acknowledged the classical view of rigidity as velocity-independent, their own data suggested otherwise, particularly when contrasted with spasticity. In sum, the preponderance of evidence supports the conclusion that rigidity in PD is not strictly velocity-independent. Rather, it encompasses both velocity-dependent and -independent components, with the former becoming increasingly evident under controlled, quantitative assessment conditions.

Parkinsonian rigidity may result from abnormal activity in the nervous system. As it has been showed, this rigidity (neural component) might be assessed using fast passive limb movements, which help reveal its velocity-dependent nature [23]. Therefore, the traditional view that Parkinsonian rigidity is velocity-independent may stem from assessments performed at low speeds, where tissue stiffness (non-neural component) predominates and behaves in a velocity-independent manner—thereby contributing to the potentially misleading and ‘classical’ definition of rigidity in PD.

Regarding methodological quality, most studies met essential criteria on the AXIS and STROBE checklists, particularly in terms of clearly defined objectives, description of outcomes, and use of validated instrumentation. However, limitations were noted, including the non-randomized application of exploration speeds, the use of convenience sampling, insufficient justification of sample sizes, and limited consideration of potential confounding variables such as medication state and disease duration. Only a minority of studies systematically tested patients both in “on” and “off” dopaminergic states, despite known fluctuations in rigidity with treatment, limiting the generalizability of their findings. Furthermore, heterogeneity in study designs, regions assessed, protocols and measurement outcomes prevent a comparison of findings.

### 4.1. Practical Implications

This systematic review highlights the velocity-dependent characteristics of Parkinsonian rigidity, demonstrating increased muscle tone with higher passive movement velocities across key joints, including the wrist, elbow, knee, and trunk. As a key practical implication, distinguishing between extrapyramidal and the more complex Parkinsonian rigidity may help clarify the role of non-neurological factors and enhance the practical applicability of these findings. For neuroengineering and rehabilitation applications, these findings emphasize the importance of incorporating controlled and standardized velocity protocols in the objective assessment of rigidity. The utilization of advanced measurement technologies such as isokinetic dynamometry, electromechanical devices, and combined biomechanical and neurophysiological approaches enables precise quantification and discrimination of neural versus non-neural contributions to rigidity. These insights facilitate the development of targeted rehabilitation strategies and assist in optimizing therapeutic interventions based on specific velocity-dependent motor impairments.

An important clinical consideration is the distinction between rigidity and spasticity when performing passive movement at different speeds. Although increased resistance during faster passive movement may suggest speed dependence, this characteristic alone is not sufficient to differentiate between the two phenomena. The presence of a speed-dependent stretch reflex could be a key criterion for identifying spasticity, in addition to other neurophysiological studies that could be implemented, whereas rigidity usually occurs without reflex activation. Therefore, clinical evaluation should combine variation in movement speed with neurophysiological assessment to ensure accurate distinction between the two phenomena.

A key strength identified in this review is the growing use of quantitative biomechanical and neurophysiological methods to assess rigidity in PD, which enhances objectivity and reproducibility across studies. Clinically, testing rigidity at different passive movement velocities could improve diagnostic accuracy and help distinguish rigidity patterns across disease stages [38,39,40,41]. However, a major weakness lies in the heterogeneity of assessment protocols, which hinders direct comparisons. This variability also represents a threat to the development of standardized clinical tools and limits the generalizability of findings. In addition, velocity-controlled assessment protocols could improve the sensitivity of clinical trials, allowing more accurate evaluation of both pharmacological and non-pharmacological interventions. Conversely, a significant opportunity emerges from this gap: the need for an international consensus on assessment protocols. Establishing standardized methodologies would not only improve inter-study comparability but also support the advancement of translational research aimed at developing targeted interventions for Parkinsonian rigidity.

Finally, in recent years, smartphones, smartwatches, and activity trackers have shown increasing potential for the assessment and monitoring of motor symptoms in individuals with PD. Their relatively low cost, accessibility, and usability support their integration into real-world clinical practice and demonstrate validity in aiding PD management [42,43,44,45,46,47]. Future research could explore the assessment of rigidity in PD by introducing different speeds of evaluation during real-world tasks, further advancing objective and dynamic disease monitoring.

### 4.2. Limitations

This review has certain limitations that should be acknowledged. Firstly, although multiple databases were consulted and a comprehensive list of studies was reviewed, publication bias cannot be excluded, and gray literature was not considered. Secondly, due to the diversity of methodologies and measurement devices used, data synthesis was narrative rather than statistical. Finally, we cannot extrapolate our findings to other pathologies with stiffness. Moreover, some older studies lacked detailed data, which may have affected quality assessments and limited interpretation of their findings.

## 5. Conclusions

While stiffness in PD is not classically defined as velocity-dependent, most evidence suggests that, under specific test conditions, stiffness increases with passive movement velocity. These findings highlight the need to reconsider traditional clinical assumptions and standardize measurement protocols to refine the understanding and assessment of stiffness in PD.

## Figures and Tables

**Table 1:** Summary of the main findings from the included studies.

Author(s), Year	Population	Clinical Characteristics	Technology Employed	Exploration Protocol	Main Findings
Teräväinen et al., 1989 [13]	PD patients (*n* = 20), Controls (*n* = 12)	H&Y stages were not reported. The patients were treated with a variety of antiparkinsonian drugs including levodopa. Many of them experienced fluctuations of disability.	Electromechanical apparatus	Passive wrist movements angular velocities from 12 to 240 degrees per second and over angular displacements from ±15 to ±30 degrees.	Objective rigidity was more pronounced at faster movement velocities in Parkinson’s patients, whereas normal subjects showed only a modest score increase. Angular velocities of 140–190°/s and displacements of ±25–30° were most sensitive for detecting Parkinsonian rigidity and correlated well with the clinical rigidity score.
Fung et al., 2000 [22]	PD patients (*n* = 20), Controls (*n* = 10)	H&Y stages (on) ranged from 0 to 4. H&Y stages (off) ranged from 1 to 5. Disease duration ranged from 5 to 30 years.	Electromechanical system + Surface electromyographic (EMG)	Passive wrist movement. The torque motor was operated as a position servo7 to generate sinusoidal perturbations of 60° peak-to-peak amplitude about the 0° position (that is, ±30°)	Angular impulse is a valid objective measure of Parkinsonian rigidity. Activation increases rigidity, but to varying degrees across patients. To improve the sensitivity and reproducibility of clinical rigidity assessments, Parkinson’s rating scales should include separate resting and activated scores. Both elastic and non-elastic forces contribute to the clinical perception of Parkinsonian rigidity.
Asci et al., 2023 [23]	PD patients (*n* = 20), Controls (*n* = 25)	PD (67.3 ± 6.9 years). 25 age- and sex-matched controls (66.9 ± 7.4 years). Hoehn & Yahr stage 2.1 ± 0.6. UPDRS-III: 21.8 ± 8.2. Upper limb rigidity in UPDRS-III: 2.1 ± 0.8 Disease duration 5.1 ± 2.5 years.	Biomechanical device + EMG	Wrist extensions at seven different angular velocities (5–50–100–150–200–236–280°/s) randomly applied, when ON therapy. Range of motion (ROM) of 50° (i.e., ranging from −20° to +30°).	In patients, objective rigidity progressively increased with rising angular velocities during robot-assisted wrist extensions. Neurophysiological examination revealed increased long-latency reflexes, but not short-latency reflexes or the shortening reaction, in PD compared with control subjects. Long-latency reflexes increased progressively with angular velocity only in people with PD. Specific biomechanical and neurophysiological abnormalities correlated with the clinical rigidity score.
Powell et al., 2012 [24]	PD patients (*n* = 18)	64.1 ± 9.0 years old. Disease duration ranged from 1 to 14 years. Rigidity (in UPDRS) ranged from 0 to 3 (on) and from 2 to 3 (off).	Electromechanical device + EMG	Passive wrist movement through 60° and 90° ranges of wrist flexion and extension at velocities of 50°/s and 280°/s in both treated and untreated conditions.	Both work scores and angular impulses showed that larger displacement amplitudes and higher velocities were associated with significantly greater rigidity, increased EMG ratio, and higher mean EMG of stretched muscles. Dopaminergic medication was not associated with a reduction in rigidity. Parkinsonian rigidity is modulated by the amplitude and rate of muscle stretch.
Zetterberg et al., 2015 [25]	PD patients (*n* = 25), Controls (*n* = 14)	Mean age in PD group was 72 ± 5.9 (SD) years, mean time since diagnosis was 7 ± 5.3 (SD) years, and mean age for controls was 73 ± 4.9 (SD) years.	NeuroFlexor device	Passive wrist movements. The range of wrist movement was 50°, starting position at 20° flexion and end position at 30° extension. Two velocities were used: slow 5°/s and fast 236°/s.	The findings suggest that stretch-induced reflex activity, rather than non-neural resistance, is the major contributor to rigidity in wrist muscles in PD. The NeuroFlexor is a potentially valuable clinical and research tool for the quantification of rigidity.
Xia et al., 2016 [26]	PD patients (*n* = 14), Controls (*n* = 14)	The average ages were 62.6 (±9.1) years for the PD group and 62.9 (±8.5) years for the control group. H&Y ranged from 1 to 4. UPDRS (rigidity) ranged from 0 to 3 (on); and from 2 to 3 (off). Disease duration (years) ranged from 1 to 13.	Electromechanical + EMG	Passive wrist movement at 50º/s. Each subject with PD was tested in Off- and On-medication states.	The results showed that the reflex and intrinsic components were comparable (*p* > 0.05), and both were greater in PD subjects than in controls (*p* < 0.05). Medication decreased the reflex component of stiffness (*p* < 0.01). These findings indicate that both reflex and intrinsic factors contribute to stiffness.
Xia et al., 2009 [27]	PD patients (*n* = 20)	Subjects’ age ranged from 52 to 74 years with an average of 64 ± 7 years. Disease duration (years) ranged from 1 to 11. UPDRS (rigidity) ranged from 0 to 2 (on); and from 2 to 3 (off).	Electromechanical device + EMG	Wrist flexion and extension within ±30º at velocities 50 and 280/s. PD patients were evaluated in Off-medication and On medication states	Both the stretch reflex and the shortening reaction are important determinants of rigidity.
Zito et al., 2018 [28]	PD patients (*n* = 4), Controls (*n* = 12)	People with PD with a mean age = 50.5 years, SD = 4.2. No more clinical data were showed.	Novel electromechanical wrist device	Passive wrist movement at velocities of 10°/s, 50°/s, and 100°/s. From 7° to −37°.	The comparison of median rigidity between PD patients and HC showed significant differences at all tested velocities, during both flexion and extension. These findings are consistent with the literature, which indicates that the main contributor to rigidity in PD is a stretch-reflex-induced increase in passive movement resistance.
Lee et al., 2002 [14]	Stroke patients (*n* = 12), PD patients (n= 16), Controls (*n* = 12)	Twelve subjects had spastic hemiparesis, age range 31 to 77 years (mean (SD), 62.2 (6.5) years), with time from onset varying from 8 weeks to 20 years. Sixteen had rigid parkinsonism, age range 27 to 85 years (mean 60.6 (13.5)) and time of onset between 4 weeks and 6 years. The control group consisted of 12 age matched normal persons without a history of neurological disease.	Electromechanical apparatus	Elbow flexors were vertically stretched under four different velocities (40, 80, 120, and 160°/s) through a 75 degrees range of motion.	Velocity-dependence analysis indicates that rigidity and spasticity have approximately equal velocity-dependent properties. To differentiate these two types of hypertonia, position-dependent properties may be used. However, the progressively increasing muscle tone of spasticity differs from the increased (relative to normal) but constant muscle tone observed in parkinsonism.
Endo et al., 2015 [29]	PD patients (*n* = 20)	Mean age: 74.4 ± 6.2 years. The UPDRS rigidity score was 1 in 8 patients, 2 in 9 patients, and 3 in 3 patients.	Muscle tone measurement device.	Passive elbow movement from 10 to 110º at two different velocities, 60°/s and 120°/s. All patients were on medication during the measurements.	The features of rigidity may differ from the conventional definition, which states that rigidity is not dependent on the velocity of joint movement.
Huang et al., 2016 [32]	Controls (*n* = 22), Stoke patients (*n* = 14), PD patients (*n* = 21)	Controls: 63.2 (7.5) years. PD: 68.3 (9.9) years. Stroke: 65.1 (8.8) years.	Pendulum test	Elbow joint passive oscillations at spontaneous frequency	No differences were observed between these two patient groups. Hypertonia in Parkinsonian and stroke patients could not be differentiated by the modified pendulum test; elbow extensors exhibited higher muscle tone in both control and patient groups; and hypertonia in both Parkinsonian and stroke patients was velocity dependent.
Rothwell et al., 1983 [16]	PD patients (*n* = 47), Controls (*n* = 12)	The patients were classified clinically into four groups according to the degree of rigidity at the elbow or tremor.	Electromechanical apparatus + EMG	Upper limb: Long latency stretch reflexes in flexor pollicis longus and triceps brachii was assessed. Six velocities of ramp were used from 15°/s to 600°/s, reaching a maximum displacement of 20º.	Enhanced long-latency stretch reflexes contribute to, but may not be solely responsible for, rigidity in Parkinson’s disease. This technique revealed increased reflex sensitivity of the flexor pollicis longus in both moderately and severely rigid patients, which was not evident using step-torque stretches alone.
Nuyens et al., 2000 [30]	PD patients (*n* = 10), Controls (*n* = 10)	65.4 ± 7.41 years had a disease duration of 10 ± 5.23 years. Grades on the modified Hoehn and Yahr staging ranged between 2 and 5. Schwab and England scores varied between 20 and 90%.	Isokinetic dynamometer + EMG	Knee passive flexion-extension at speeds of 60º, 180º and 300º/s in series of ten consecutive movements per velocity.	PD patients demonstrated a greater decrease in resistive torque compared with healthy controls, particularly at higher velocities and during knee flexion. These results contribute to the ongoing debate on whether Parkinsonian rigidity is independent of movement speed and direction.
Uslu et al., 2021 [31]	PD patients (*n* = 40)	Duration of the disease was 5.5 ± 0.67 years. The number of participants in the subgroups was 8 (4 male, 4 female) for the UPDRS 1 group, 16 (11 male, 5 female) for the UPDRS 2 group, 11 (5 male, 6 female) for the UPDRS 3 group and 5 (2 male, 3 female) for the UPDRS 4 group. H&Y ranged from 1 to 4.	Pendulum test + EMG	Knee reflex triggered pendulum at different amplitudes and velocities.	Parkinson’s rigidity has a velocity-dependent component, which correlates negatively with the rigidity scale.
Gregoric et al., 1988 [33]	PD patients (*n* = 7)	--	Electromechanical apparatus	Rigidity was measured during sinusoidal passive movements of the ankle joint	Velocity-dependent changes were observed, though less pronounced than in spasticity and expressed differently in flexor and extensor muscles: a mild decrease in resistive torque during faster stretching of dorsal flexors and an increase in resistance during stretching of plantar flexors. Dorsal flexors also frequently exhibited shortening reactions.
Mak et al., 2007 [15]	PD patients (*n* = 15), Controls (*n* = 15)	PD: mean age 64.7 (8.7) years. Hoehn and Yahr staging scores between 2–3	Isokinetic dynamometry	Trunk movements at 60/s, 75/s, 90/s and 105/s. All tests were performed within 2 h after medication during the ‘‘on’’ phase of the medication cycle.	PD patients exhibited significantly higher muscle tone, indicated by increases in work done and torque at higher movement speeds. Within each subject group, resistive trunk muscle tone increased with passive movement velocity, but the increase was greater in PD patients. Rigidity is therefore not independent of movement speed.
Cano-de-la-Cuerda et al., 2014 [34]	PD patients (*n* = 36)	The mean age of the sample was 62 ± 11 years. Overall, 24 patients were classified as II, 8 as IB (unilateral and axial involvement) and 4 as III in the H&Y staging score. The mean value of disease severity in the UPDRS III was 22 ± 8 points. A total of 26 subjects reached 80%, 7 reached 90% and 3 reached 100% in the Schwab and England scale. The mean disease duration was 55.4 ± 14.3 months	Isokinetic dynamometry	Trunk flexion-extension at 30°/s, 45°/s, 60°/s	Their results suggested that angular velocities of 30°/s, 45°/s, and 60°/s using this objective method provided a valid assessment of trunk rigidity and correlated with disease severity, disease duration, functional status, and quality of life in PD patients. Rigidity appears to increase with assessment speed.

**Table 2:** Appraisal Tool for Cross-Sectional Studies (AXIS).

AXIS Item/Study	1	2	3	4	5	6	7	8	9	10	11	12	13	14	15	16	17	18	19	20
Teräväinen et al. 1989 [13]	✓	?	✗	✓	✗	✗	✗	✓	✗	✓	✓	✗	✗	✗	✓	✗	✓	?	?	✗
Fung et al. 2000 [22]	✓	?	✗	✓	✓	✓	✗	✓	✗	✓	✓	✗	✗	✗	✓	✗	✓	?	?	✗
Asci et al. 2023 [23]	✓	✓	✓	✓	✓	✓	✓	✓	✓	✓	✓	✓	✓	✓	✓	✓	✓	✓	✓	✓
Powell et al. 2012 [24]	✓	?	✗	✓	✓	✓	✗	✓	✗	✓	✓	✗	✗	✗	✓	✗	✓	?	?	✗
Zetterberg et al. 2015 [25]	✓	✓	✓	✓	✓	✓	✓	✓	✓	✓	✓	✓	✓	✓	✓	✓	✓	✓	✓	✓
Xia et al. 2016 [26]	✓	✓	✓	✓	✓	✓	✓	✓	✓	✓	✓	✓	✓	✓	✓	✓	✓	✓	✓	✓
Xia et al. 2009 [27]	✓	✓	✓	✓	✓	✓	✓	✓	✓	✓	✓	✓	✓	✓	✓	✓	✓	✓	✓	✓
Zito et al. 2018 [28]	✓	✓	✓	✓	✓	✓	✓	✓	✓	✓	✓	✓	✓	✓	✓	✓	✓	✓	✓	✓
Lee et al. 2002 [14]	✓	?	✗	✓	✓	✓	✗	✓	✗	✓	✓	✗	✗	✗	✓	✗	✓	?	?	✗
Endo et al. 2015 [29]	✓	✓	✓	✓	✓	✓	✓	✓	✓	✓	✓	✓	✓	✓	✓	✓	✓	✓	✓	✓
Rothwell et al. 1983 [16]	✓	?	✗	✓	✗	✗	✗	✓	✗	✓	✓	✗	✗	✗	✓	✗	✓	?	?	✗
Nuyens et al. 2000 [30]	✓	?	✗	✓	✓	✓	✗	✓	✗	✓	✓	✗	✗	✗	✓	✗	✓	?	?	✗
Uslu et al. 2021 [31]	✓	✓	✓	✓	✓	✓	✓	✓	✓	✓	✓	✓	✓	✓	✓	✓	✓	✓	✓	✓
Huang et al. 2016 [32]	✓	✓	✓	✓	✓	✓	✓	✓	✓	✓	✓	✓	✓	✓	✓	✓	✓	✓	✓	✓
Gregoric et al. 1998 [33]	✓	?	✗	✓	✗	✗	✗	✓	✗	✓	✓	✗	✗	✗	✓	✗	✓	?	?	✗
Mak et al. 2007 [15]	✓	✓	✓	✓	✓	✓	✓	✓	✓	✓	✓	✓	✓	✓	✓	✓	✓	✓	✓	✓
Cano-de-la-Cuerda et al. 2014 [34]	✓	✓	✓	✓	✓	✓	✓	✓	✓	✓	✓	✓	✓	✓	✓	✓	✓	✓	✓	✓

✓ = Yes/Reported/Adequate; ✗ = No/Not Reported/Inadequate; ? = Unclear/Not specified. 1. Were the aims/objectives of the study clear? 2. Was the study design appropriate for the stated aim(s)? 3. Was the sample size justified? 4. Was the target/reference population clearly defined? (Is it clear who the research was about?) 5. Was the sample frame taken from an appropriate population base so that it closely represented the target/reference population under investigation? 6. Was the selection process likely to select subjects/participants that were representative of the target/reference population under investigation? 7. Were measures undertaken to address and categorize non-responders? 8. Were the risk factor and outcome variables measured appropriate to the aims of the study? 9. Were the risk factor and outcome variables measured correctly using instruments/measurements that had been trailed, piloted, or published previously? 10. Is it clear what was used to determine statistical significance and/or precision estimates? (e.g., *p*-values, confidence intervals) 11. Were the methods (including statistical methods) sufficiently described to enable them to be repeated? 12. Were the basic data adequately described? 13. Does the response rate raise concerns about non-response bias? 14. If appropriate, was information about non-responders described? 15. Were the results internally consistent? 16. Were the results presented for all the analyses described in the methods? 17. Were the authors’ discussions and conclusions justified by the results? 18. Were the limitations of the study discussed? 19. Were there any funding sources or conflicts of interest that may affect the authors’ interpretation of the results? 20. Was ethical approval or consent of participants attained?.

**Table 3:** Checklist of Strengthening the Reporting of Observational Studies in Epidemiology (STROBE) guidelines.

	STROBE Item	Teräväinen et al., 1989 [13]	Fung et al., 2000 [22]	Asci et al., 2023 [23]	Powell et al., 2012 [24]	Zetterberg et al., 2015 [25]	Xia et al., 2009 [27]	Xia et al., 2016 [26]	Zito et al., 2018 [28]	Lee et al., 2002 [14]	Endo et al., 2015 [29]	Rothwell et al., 1983 [16]	Nuyens et al., 2000 [30]	Uslu et al., 2021 [31]	Huang et al., 2016 [32]	Gregoric et al., 1998 [33]	Mak et al., 2007 [15]	Cano-de-la-Cuerda et al., 2014 [34]
1a	Design named in title/abstract	✓	✓	✓	✓	✓	✓	✓	✓	✓	✓	✓	✓	✓	✓	✓	✓	✓
1b	Structured abstract summary	✓	✓	✓	✓	✓	✓	✓	⚠	✓	✓	✓	⚠	✓	✓	⚠	✓	⚠
2	Background/rationale	✓	✓	✓	✓	✓	✓	✓	✓	✓	✓	✓	✓	✓	✓	✓	✓	✓
3	Objectives (with hypotheses)	✓	✓	✓	✓	✓	✓	✓	✓	✓	✓	✓	✓	✓	✓	✓	✓	✓
4	Design described early	✓	✓	✓	✓	✓	✓	✓	✓	✓	✓	✓	✓	✓	✓	✓	✓	✓
5	Setting & dates	⚠	⚠	✓	⚠	✓	⚠	✓	⚠	⚠	✓	⚠	⚠	✓	✓	⚠	✓	✓
6a	Eligibility & selection criteria	⚠	⚠	✓	⚠	✓	✓	✓	✓	✓	✓	⚠	⚠	✓	⚠	⚠	✓	✓
7	Defined variables (exposure/outcome/confounders)	✓	✓	✓	✓	✓	✓	✓	⚠	✓	✓	✓	✓	✓	✓	✓	✓	✓
8	Data sources/measurement methods	✓	✓	✓	✓	✓	✓	✓	⚠	✓	✓	✓	✓	✓	✓	✓	✓	✓
9	Efforts to address bias	⚠	⚠	✓	⚠	✓	✓	✓	⚠	⚠	✓	⚠	⚠	⚠	⚠	⚠	⚠	⚠
10	Sample size justification	✗	⚠	✓	⚠	✓	✓	✓	⚠	⚠	✓	✗	⚠	✓	⚠	✗	✓	✓
11	Handling of quantitative variables	⚠	⚠	✓	⚠	✓	✓	✓	⚠	⚠	✓	⚠	⚠	✓	⚠	⚠	✓	✓
12a	Statistical methods, including confounding	⚠	⚠	✓	⚠	✓	✓	✓	⚠	⚠	✓	⚠	⚠	✓	⚠	⚠	✓	✓
12b	Subgroup/interactions methods	✗	✗	✓	✗	✓	✓	✓	✗	✗	✓	✗	✗	✓	✗	✗	✓	✓
12c	Methods for missing data	✗	✗	✓	✗	✓	✓	✓	✗	✗	✓	✗	✗	✓	✗	✗	✓	✓
12d	Accounting for sampling strategy	✗	✗	✓	✗	✓	✓	✓	✗	✗	✓	✗	✗	✓	✗	✗	✓	✓
12e	Sensitivity analyses	✗	✗	⚠	✗	⚠	✓	✓	✗	✗	✗	✗	✗	✓	✗	✗	✓	✓
13a	Participant flow numbers	✗	⚠	✓	✗	✓	✗	✗	✗	⚠	✓	⚠	⚠	✓	✓	⚠	✓	✓
13b	Reasons for non-participation	✗	✗	✓	✗	✓	✗	✗	✗	✗	✓	✗	✗	✓	✓	✗	✓	✓
13c	Flow diagram considered	✗	✗	⚠	✗	✓	✗	✗	✗	✗	✓	✗	✗	✓	✗	✗	✓	✓
14a	Descriptive data (participant characteristics)	✓	✓	✓	✓	✓	✓	✓	⚠	⚠	✓	⚠	⚠	✓	✓	⚠	✓	✓
14b	Missing data per variable	✗	✗	✓	✗	✓	✗	✗	✗	✗	✓	✗	✗	✓	✓	✗	✓	✓
15	Outcome data (events/summary measures)	✓	✓	✓	✓	✓	✓	✓	⚠	✓	✓	✓	✓	✓	✓	⚠	✓	✓
16a	Unadjusted & adjusted estimates	⚠	⚠	✓	⚠	✓	✓	✓	⚠	✓	✓	⚠	⚠	✓	✓	⚠	✓	✓
16b	Report variable categorization boundaries	✗	✗	✓	✗	✓	✓	✓	✗	✗	✓	✗	✗	✓	✗	✗	✓	✓
16c	Translate relative to absolute risk	✗	✗	⚠	✗	⚠	⚠	⚠	✗	✗	⚠	✗	✗	⚠	✗	✗	⚠	⚠
17	Other analyses (subgroups/sensitivity)	✗	✗	✓	✗	✓	✓	✓	✗	✗	✓	✗	✗	✓	✗	✗	✓	✓
18	Key results summary	✓	✓	✓	✓	✓	✓	✓	✓	✓	✓	✓	✓	✓	✓	✓	✓	✓
19	Limitations discussed	⚠	⚠	✓	⚠	✓	✓	✓	⚠	⚠	✓	⚠	⚠	✓	✓	⚠	✓	✓
20	Interpretation in context	✓	✓	✓	✓	✓	✓	✓	✓	✓	✓	✓	✓	✓	✓	✓	✓	✓
21	Generalizability discussed	⚠	⚠	✓	⚠	✓	✓	✓	⚠	⚠	✓	⚠	⚠	✓	⚠	⚠	✓	✓
22	Funding and role of funders disclosed	✗	✗	✓	✗	✓	✓	✓	✗	✗	✓	✗	✗	✓	✗	✗	✓	✓

✓: Item is adequately reported; ⚠: Item is partially reported or unclear; ✗: Item is not reported.

**Table 4:** Strengths, Weaknesses, Opportunities and Threats (SWOT) Analysis of the Included Studies.

Strengths	Weaknesses
Use of Advanced Instrumentation: Many studies employed sophisticated biomechanical and neurophysiological tools (e.g., isokinetic dynamometry, NeuroFlexor, EMG-integrated devices), allowing precise quantification of passive resistance and its components. Inclusion of Multiple Joints: The studies covered a broad range of anatomical regions (wrist, elbow, knee, trunk), providing a more comprehensive understanding of rigidity expression across the body. Objective and Quantitative Approaches: Most articles moved beyond subjective clinical scales, using measurable biomechanical indices (e.g., torque, work, angular impulse) to capture rigidity changes with velocity. Recent High-Quality Studies: More recent contributions (e.g., Asci et al., 2023) [23] demonstrated excellent methodological transparency and addressed both reflexive and non-reflexive mechanisms underlying rigidity.	Protocol Heterogeneity: There was substantial variability in passive movement velocities, durations, angles, and devices used, making inter-study comparisons difficult and precluding meta-analysis. Small Sample Sizes: Several studies had limited participant numbers without clear sample size justification, reducing statistical power and generalizability. Incomplete Reporting: Some studies did not fully meet reporting standards (e.g., missing participant flow, recruitment strategy, or blinding), as identified in AXIS and STROBE assessments. Lack of Standardization in Medication State: Medication status (“on” vs. “off” levodopa) was not consistently controlled or reported, even though it significantly influences rigidity.
**Opportunities**	**Threats**
Development of Standardized Protocols: Future studies can build consensus on optimal joint angles, velocities, and outcome measures to assess rigidity and its velocity dependency consistently. Integration of Multimodal Measurements: Combining mechanical, EMG, and neuroimaging data could help disentangle the neural vs. non-neural contributions to rigidity and explore its pathophysiological basis more deeply. Clinical Translation: Rigidity metrics that are velocity-sensitive may aid in differential diagnosis (e.g., spasticity vs. rigidity) or in tracking treatment responses in PD and atypical parkinsonism. Longitudinal Studies: Most current studies are cross-sectional. Longitudinal work could help understand how rigidity and its velocity-dependence evolve with disease progression or treatment.	Technological Accessibility: High-end measurement tools used in some studies may not be widely available in clinical settings, limiting their translational impact. Diagnostic Overlap: In older patients or those with overlapping motor syndromes (e.g., PSP), distinguishing between rigidity and spasticity remains challenging, especially in biomechanical tests alone. Lack of Consensus on Terminology: The inconsistency in how rigidity, tone, and stiffness are defined or operationalized may obscure the interpretation and comparison of findings. Potential Bias in Older Studies: Several early publications lacked methodological rigor or failed to use blinded assessors or validated instruments, which may bias their conclusions.

## Data Availability

The datasets analyzed during the current study are available from the corresponding author upon request. Please contact Roberto Cano de la Cuerda. (roberto.cano@urjc.es).

## Supplementary Materials

The following supporting information can be downloaded at: https://www.mdpi.com/article/10.3390/neurolint17110186/s1, Table S1: PRISMA list.

## Abbreviations

The following abbreviations are used in this manuscript:

AXIS	Appraisal Tool for Cross-Sectional Studies
EMG	Electromyography
H&Y	Hoehn and Yahr
LLSR	Long-latency stretch reflex
PD	Parkinson’s disease
PRISMA	Preferred Reporting Items for Systematic Reviews and Meta-Analyses
PROSPERO	International Prospective Register of Systematic Reviews
ROM	Range of motion
SD	Standard deviation
SPASM	Support Programme for Assembly of a Database for Spasticity Measurement
STROBE	Strengthening the Reporting of Observational Studies in Epidemiology
UPDRS	Unified Parkinson’s Disease Rating Scale
